# Salinity modulates thermotolerance, energy metabolism and stress response in amphipods *Gammarus lacustris*

**DOI:** 10.7717/peerj.2657

**Published:** 2016-11-17

**Authors:** Kseniya P. Vereshchagina, Yulia A. Lubyaga, Zhanna Shatilina, Daria Bedulina, Anton Gurkov, Denis V. Axenov-Gribanov, Boris Baduev, Elizaveta S. Kondrateva, Mikhail Gubanov, Egor Zadereev, Inna Sokolova, Maxim Timofeyev

**Affiliations:** 1 Institute of Biology, Irkutsk State University, Irkutsk, Russia; 2 Baikal Research Centre, Irkutsk, Russia; 3 Institute of Biophysics SB RAS, Krasnoyarsk, Russia; 4 Siberian Federal University, Krasnoyarsk, Russia; 5 Institute for Biological Sciences, University of Rostock, Rostock, Germany

**Keywords:** Thermal tolerance, Salinity, *Gammarus lacustris*, Adaptation, Amphipoda

## Abstract

Temperature and salinity are important abiotic factors for aquatic invertebrates. We investigated the influence of different salinity regimes on thermotolerance, energy metabolism and cellular stress defense mechanisms in amphipods *Gammarus lacustris* Sars from two populations. We exposed amphipods to different thermal scenarios and determined their survival as well as activity of major antioxidant enzymes (peroxidase, catalase, glutathione S-transferase) and parameters of energy metabolism (content of glucose, glycogen, ATP, ADP, AMP and lactate). Amphipods from a freshwater population were more sensitive to the thermal challenge, showing higher mortality during acute and gradual temperature change compared to their counterparts from a saline lake. A more thermotolerant population from a saline lake had high activity of antioxidant enzymes. The energy limitations of the freshwater population (indicated by low baseline glucose levels, downward shift of the critical temperature of aerobic metabolism and inability to maintain steady-state ATP levels during warming) was observed, possibly reflecting a trade-off between the energy demands for osmoregulation under the hypo-osmotic condition of a freshwater environment and protection against temperature stress.

## Introduction

Temperature and salinity are important abiotic factors affecting survival and performance of aquatic invertebrates and setting limits to their geographical distribution (Kinne, Schmitz & Kinne-Saffran, 1971; Nelson, Fatland & Cardwell, 1977; Pörtner & Farrell, 2008). Recent data indicate the effect of the global climate change to surface waters including lakes worldwide (O’Reilly et al., 2015). The bulk of aquatic organisms are ectothermic, that makes them particularly responsive to one of the main effects of global climate change: the increased variability in temperature, due to the dependency of their body temperature and the metabolic processes from the ambient water temperature. Temperature strongly affects ectotherms due to its direct effects on the rates of physiological and biochemical reactions (Hochachka, Fields & Mustafa, 1973; Cossins, Schwarzbaum & Wieser, 1995; Somero, 2004). Temperature, fluctuations often co-occur with other selective pressures, such as variations in the salt content of the water. In aquatic organisms, deviations from the species-specific optimal salinity can result in osmotic stress requiring adjustments of the cell volume and modulation of enzyme activity to maintain normal cellular functions under the altered ionic conditions; these adjustments can lead to elevated energy demand for osmotic, ionic and/or cell volume regulation (Glazier & Sparks, 1997; Kinne, Schmitz & Kinne-Saffran, 1971; Freiere et al., 2011). Therefore, environmental stress (including salinity and temperature stress) can significantly influence the energy balance of living organisms due to the additional energy required to restore and maintain homeostasis, which can put a strain on the energy acquisition, transformation and conservation systems (Sokolova et al., 2012; Acker, 1988; Calow & Forbes, 1998; Amiard-Triquet, Rainbow & Roméo, 2011).

Antioxidant systems play a major role in environmental stress response. Antioxidants maintain the cellular redox balance and prevent excess of reactive oxygen species (ROS) from interacting with the critical intracellular structures (Circu & Aw, 2010; Jones, 2008). Antioxidant responses are involved in responses to thermal stress in aquatic organisms (Abele et al., 2007; Sokolova, Sukhotin & Lannig, 2011), and earlier studies suggest that some stressors (such as trace metals and hypoxia) that affect cellular redox balance, can modulate the cellular response to thermal stress in aquatic organisms (Ivanina, Taylor & Sokolova, 2009; Pörtner, Langenbuch & Michaelidis, 2005; Sokolova & Lannig, 2008). However, the effects of environmental salinity on thermotolerance and cellular protection mechanisms of aquatic organisms are not well understood and require further investigation.

Freshwater amphipods of the genus *Gammarus* Fabricius, 1775 are a useful model to study the interactive effects of salinity and thermal stress. *Gammarus* is a widespread genus of amphipods in the northern hemisphere that plays a key role in the food webs of freshwater ecosystems. Some freshwaters amphipods (including those of genus *Gammarus*) occur in habitats ranging from low mineralization fresh waters to brackish waters. These species also experience wide diurnal and seasonal temperature fluctuations in temperate shallow waters (Jacobs et al., 1997) and thus must possess efficient mechanisms of protection against temperature-induced cellular stress. The molecular and cellular mechanisms of the broad salinity and temperature tolerance of freshwater amphipods remain to be fully elucidated (Grzesiuk & Mikulski, 2006).

The aim of this study was to determine whether acclimation/adaptation to habitats with different salinity regimes modulates the energy metabolism, cellular protective responses to temperature stress and thermal tolerance of amphipod *Gammarus lacustris* Sars (Sars, 1863). We exposed amphipods from two populations adapted to different salinity regimes to several thermal scenarios (namely hypothermia, as well as acute and gradual warming) and determined their survival, activity of major antioxidant enzymes (peroxidase, catalase, glutathione S-transferase) and parameters of energetic metabolism (content of glucose, glycogen, ATP, ADP, AMP and lactate) in order to gain insights into the physiological and cellular mechanisms of temperature-salinity interactions in this ecologically important eurybiont species.

## Materials and Methods

### Animals

Holarctic amphipod *Gammarus lacustris* Sars, 1863 is a widespread omnivore species that inhabits lentic and lotic ecosystems and has a broad tolerance to environmental stressors (Karaman & Pinkster, 1977; Barnard & Barnard, 1983; Matafonov, 2007; Väinölä et al., 2007; Takhteev, Berezina & Sidorov, 2015). This species reproduces during the summer and can have several reproduction periods depending on the environmental and ecological characteristics of the water bodies (Timoshkin et al., 2001; Timoshkin et al., 2008). Under the experimental conditions the preferred temperatures for *G. lacustris* is 15–16 °C (Timofeyev, 2010). It is a euryhaline species with a broad pH tolerance (6.2–9.2) that inhabits benthic and pelagic zones of the lakes and is often the top predator in the absence of fish (Wilhelm & Schindler, 1999; Matafonov, 2007).

### Sampling locations

We collected amphipods in July of 2009, 2013 and 2014 from two different habitats (a freshwater habitat and a saline lake) within Eastern and Western Siberia (Russia). A freshwater population of *G. lacustris* was collected from a shallow lake formed by a backwater of Angara River in the urban area of Irkutsk city (52°16^′^4.71″N, 104°16^′^52.77″E). Amphipods were sampled by a hand net from the depth 0–1 m. Water samples were collected at the same time and analyzed for ionic composition by the Interinstitutional Regional Laboratory of Environmental Research at Irkutsk State University. Specimens of amphipods from a saltwater population were obtained from the southern shore of a meromictic lake Shira (54°29^′^7.25″N, 90°12^′^1.49″E) from the depth of 7 m using a plankton net. The freshwater site has pH 8.4 and low mineralization (0.5 g L^−1^) of the water (Table 1). The saline lake has a high content of dissolved minerals (15–17 g l^−1^) with Na^+^, Mg^2+^, and Ca^2+^ as the major cations (Kalacheva et al., 2002) (Table 1). Thermal regimes are similar at the two study sites. Both lakes can completely freeze in winter, and summer temperature may reach 23 °C in the near-shore waters. Annual average temperature in both waterbodies is 6–7 °C (Rogozin et al., 2010).

**Table 1:** Chemical composition of the surface water from brackish Lake Shira and a freshwater lake in Irkutsk (Data for Lake Shira from Kalacheva et al., 2002).

Lake	Ions	Cl^−^	Na^+^	K^+^	Mg^2+^	CO${}_{3}^{2-}$	Ca^2+^	SO${}_{4}^{2-}$	HCO${}_{3}^{-}$	pH	Mineralization g/L
Shira	mg/L	2100	2880	37	1080	174	51	8010	998	8.7	16.60
In Irkutsk		55.8	47.6	1.6	35.7	15	48.1	105	223	8.4	0.53

### Experimental design

Two types of experiments were carried out in this study: an acute temperature stress and gradual warming. Prior to the exposures animals were pre-acclimated for 3–7 days under the constant aeration in filtered water from their native habitats. Pre-acclimation was conducted at the temperature recorded at the time of sampling (15 °C for both populations) or at 7 °C (as annual average temperature of both waterbodies) in the case of the gradual warming. Amphipods were fed daily with natural food (elm leaves) with addition of commercial food (Tetra-Min, Tetra GmbH, Germany) and potato *ad libitum*. No mortality was observed during pre-acclimation. Only actively swimming animals were used for experiments. Control animals for all experiments were kept under the same conditions as during the preliminary acclimation.

#### Mortality

To determine median lethal times (LT50) that cause 50% mortality during acute heat exposure, 10 individuals from each of the two studied populations were placed in separate tanks filled with 1 L of the filtered water from their respective habitats pre-heated to 30 °C. Nine replicate tanks were used for each of the studied populations, to the total of 90 animals per population. Mortality was monitored every hour until all animals died.

#### Acute thermal stress

Amphipods were placed in 2.5 L aerated glass tanks (*n* = 5) with water, pre-heated to 30 °C (according to our observations, the maximum temperature in the littoral zones of lake in Irkutsk can reach up to 30 °C in a scorching summer). After 0.5, 1, 3 and 6 h of exposure, three individuals from each of the five replicate tanks were collected and flash-frozen in liquid nitrogen for subsequent biochemical analyses.

#### Gradual temperature increase

Animals were pre-acclimated for seven days at the temperature of 7 °C in separate glass tanks (2.5 L, *n* = 7) and subjected to the gradual temperature increase of 1 °C per hour continuing until 100% mortality occurred (modified from Sokolova & Pörtner (2003)). After every 2 degrees of temperature increase (i.e., every 2 h), three specimens were collected from each tank of the seven replicate tanks and shock-frozen in liquid nitrogen.

### Biochemical methods

#### Enzymatic activities

Activities of total cellular peroxidases, as well as catalase and glutathione S-transferase were measured using standard spectrophotometric assays. Enzyme extraction was done as described elsewhere (Bedulina, Zimmer & Timofeyev, 2010) using a 1:3 (w:v) ratio of homogenization medium to amphipod biomass. Enzyme activities were measured at 25 °C in the supernatant using a SmartSpec Plus spectrophotometer (Bio-Rad, Hercules, CA, USA) and Cary 50 spectrophotometer (Varian, Palo, Alto, CA, USA). Total peroxidase activity in the soluble fraction was measured with 4.4 mM guaiacol as a substrate at 436 nm, pH 5, according to Bergmeyer & Bernt (1974). Catalase activity was measured with 2.25 mM hydrogen peroxide as a substrate at 240 nm, pH 7, according to Aebi (1984). Glutathione S-transferase activity was measured with 0.97 mM 1-chloro-2.4-dinitrobenzene (CDNB) as a substrate at 340 nm, pH 6.5 (Habig et al., 1974). Bradford assay was used to evaluate protein concentrations (Bradford, 1976). Enzyme activities were expressed in nKatal mg^−1^ protein. For each enzyme and experimental condition, we measured 4–9 biological replicates, each replicate consisting of the pooled tissues of two individuals.

#### Lactate content

Lactate levels were determined using the express-kit “Lactate-vital” (Vital–Diagnostics, St. Petersburg, Russia) according to Bergmeyer (1985). Absorbance was measured with a Cary 50 spectrophotometer (Varian, USA) at *λ* = 505 nm.

#### Metabolites

The levels of glucose, glycogen, ATP, ADP and AMP were measured using the NADH/ NADPH-dependent enzymatic methods at *λ* = 340 nm according to Grieshaber et al. (1994), Bergmeyer (1985) and Sokolova et al. (2012). Glycogen was hydrolyzed using the methods of Sokolova et al. (2012). Absorbance was measured with a Cary 50 spectrophotometer (Varian).

The adenylate energy charge was calculated according to Atkinson (1968), using the following equation AEC = (ATP + 0.5ADP)/(ATP + ADP + AMP).

### Statistical analyses

Mortality rates in both populations under heat shock conditions were fitted to the Weibull model (Wilson, 1994) in statistical package R (R Core Team, 2016), and LT50 values (times at which mortality of 50% individuals occurred) were derived from the build regression model:

(1) \begin{eqnarray*}m=100- \frac{100}{{e}^{( \frac{t}{p} )^{r}}} \end{eqnarray*}

*m*—cumulative mortality, %

*t*—time, h

*p* and *r*—regression coefficients.

All experiments were carried out with 3–8 biological replicates, and biochemical measurements were performed in triplicate (technical replicates) for each sample. Normality was tested by Kolmogorov–Smirnov test and the equal variance with Levene’s test. Data were analyzed statistically using one-way analysis of variance (ANOVA, general linear model), followed by Student–Newman–Keuls post hoc test. If the date distribution was not normal, Kruskal–Wallis with Dunn’s test was applied. Differences were considered significant at *p*-value <  0.05 (after corrections for multiple comparisons). Statistical analysis was carried out using the software package SigmaPlot (version 12, Systat Software Inc., USA/Canada).

## Results

### Mortality

**Figure 1:**
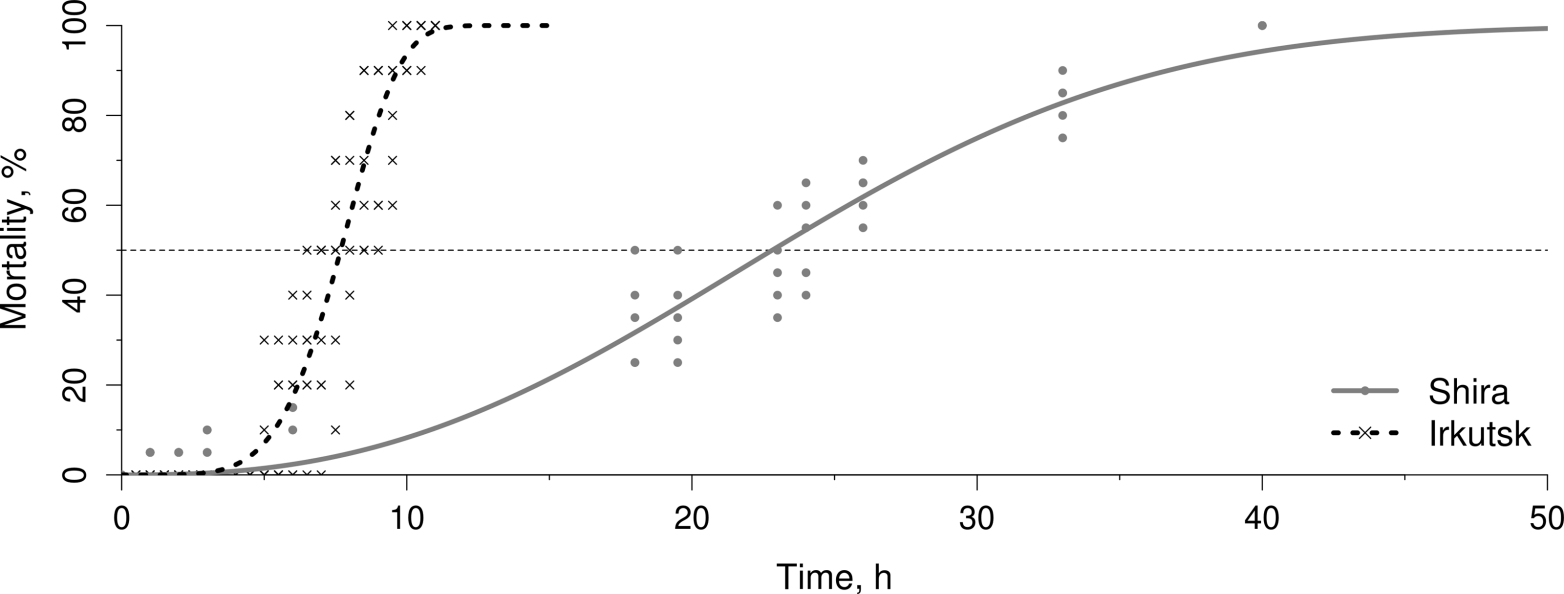
Mortality rates in Lake Shira and Irkutsk Lake populations under heat shock conditions. Data are fitted to the Weibull model.

Median mortality times (LT50) during acute exposure to 30 °C were significantly higher in amphipods from the saline Lake Shira (LT50 = 22.8 h) compared to their counterparts from a freshwater Lake in Irkutsk (LT50 = 7.7 h) (Fig. 1). A total of 100% mortality during gradual warming was observed at 31 °C in Irkutsk population and 33 °C in Shira population (data not shown).

### Activity of antioxidant enzymes

#### Peroxidase

##### Effect of acclimation at different temperatures.

**Figure 2:**
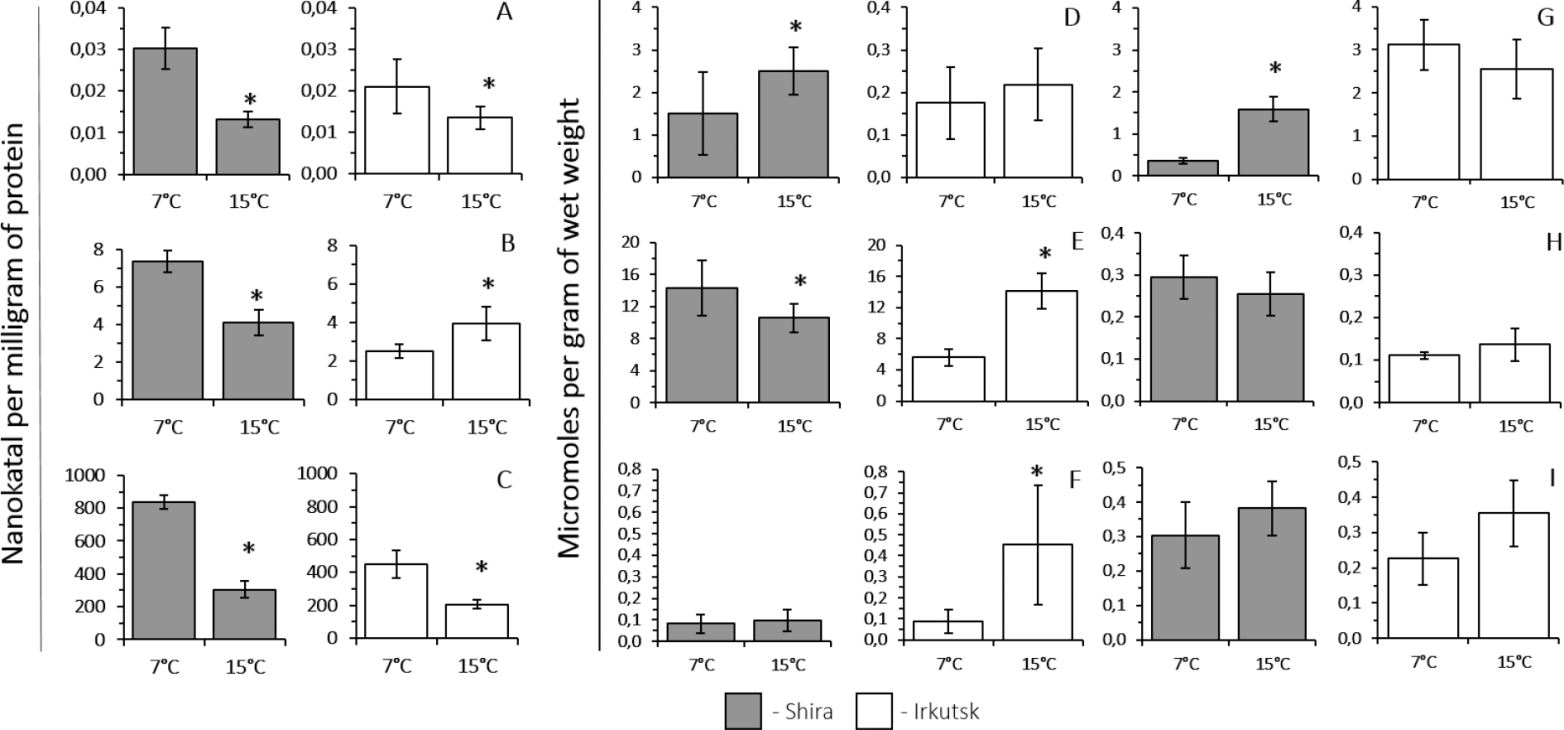
The control levels in tissues of *G. lacustris* from saltwater (Lake Shira) and freshwater (a lake in Irkutsk) populations at 7 °C and 15 °C. (A) peroxidase, (B) glutathione S-transferase, (C) catalase, (D) glucose, (E) glycogen, (F) lactate, (G) ATP, (H) AMP, (I) ADP. Asterics (*) above the columns indicate differences between populations at a given temperature (*P* < 0.05).

The levels of peroxidase activity were significantly higher after the acclimation at 7 °C than at 15 °C in amphipods from both populations (Fig. 2A).

##### Exposure to heat shock challenge.

**Figure 3:**
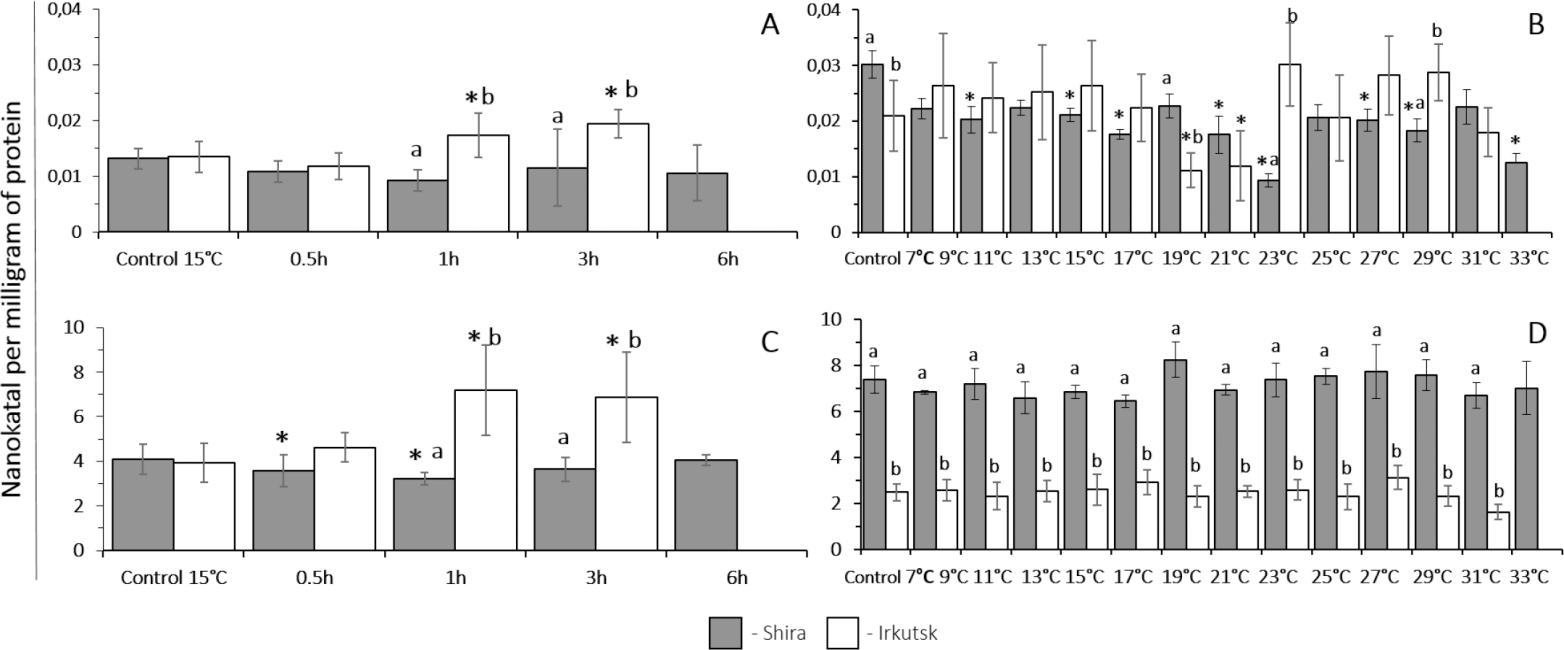
Peroxidase and glutathione S-transferase activity levels in *G. lacustris* from saltwater (Lake Shira) and freshwater (a lake in Irkutsk) populations. (A, B) peroxidase activity, (C, D) glutathione S-transferase activity. (A, C) acute exposure to 30 °C (control 15 °C). (B, D) gradual warming (1 °C h^−1^) (control 7 °C). Different letters above the columns indicate differences between populations at a given time point. Asterisks (*) denote a significant change during the thermal exposure compared to the control animals from the same population (*P* < 0.05).

The basal levels of peroxidase activity at 15 °C were similar in amphipods from the freshwater and saltwater habitats. However, in amphipods from the freshwater Irkutsk site peroxidase activity increased after 1 h of heat shock exposure (30 °C) and remained elevated until the end of the heat exposure period. In amphipods from the saltwater site, total peroxidase activity was not significantly affected by the heat shock exposure. As a result, amphipods from a freshwater Irkutsk habitat had a higher peroxidase activity during 1–3 h of heat shock exposure compared to their saltwater counterparts (Fig. 3A).

##### Exposure to the gradual thermal challenge.

The level of peroxidase activity at 7 °C was significantly higher in amphipods from the saltwater site compared to their freshwater counterparts. Beyond the exposure time, peroxidase activities changed significantly in amphipods from both populations. In the freshwater population of amphipods, peroxidase activity remained at the steady-state levels in the range of 7–17 °C and significantly decreased at 19 °C. In amphipods from Shira population, gradual warming resulted in a significant decrease of peroxidase activity at 11 °C, and peroxidase activity remained suppressed throughout the rest of the thermal exposure period (Fig. 3B).

#### Glutathione S-transferase (GST)

##### Effect of acclimation at different temperatures.

The level of GST activity was lower in amphipods from the freshwater population, acclimated at 7 °C compared to 15 °C, however in amphipods from the saltwater population the activity of this enzyme was significantly higher after the acclimation at 7 °C than at 15 °C (Fig. 2B).

##### Exposure to heat shock challenge.

The levels of GST activity at 15 °C were similar in amphipods from the freshwater and saltwater habitats. In amphipods from the freshwater Irkutsk site, there was significant increase in GST activity after 1 and 3 h of heat exposure. In amphipods from the saltwater site, heat exposure led to a transient decline in GST activity after 0.5–1 h of heat exposure, which returned the basal levels after 3–6 h of the heat shock (Fig. 3C).

##### Exposure to a gradual thermal challenge.

The baseline levels of GST activity at 7 °C were significantly higher in amphipods from the saltwater population compared to the freshwater population of amphipods. In both populations of amphipods, the activity of GST was constant throughout the exposure to gradual warming (Fig. 3D).

#### Catalase

##### Effect of acclimation at different temperatures.

The level of catalase activity was significantly higher in amphipods, acclimated at 7 °C than at 15 °C, from the both studied populations (Fig. 2C).

##### Exposure to heat shock challenge.

**Figure 4:**
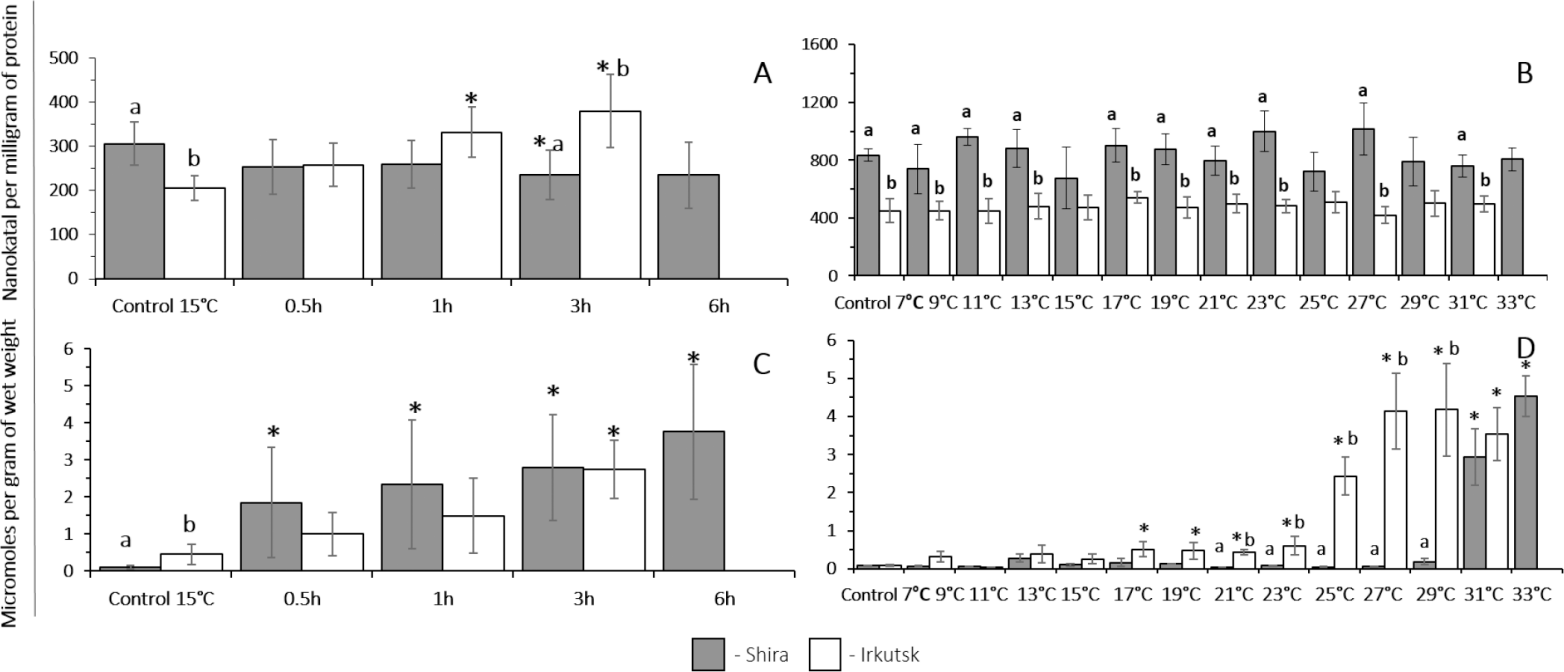
Catalase activities and lactate content in *G. lacustris* from saltwater (Lake Shira) and freshwater (a lake in Irkutsk) populations. (A, B) catalase activity, (C, D) lactate content. (A, C) acute exposure to 30 °C (control 15 °C). (B, D) gradual warming (1 °C h^−1^) (control 7 °C). Different letters above the columns indicate differences between populations at a given time point. Asterisks (*) denote a significant change during the thermal exposure compared to the control animals from the same population (*P* < 0.05).

The baseline levels of catalase activity at 15 °C were significantly higher in amphipods from the saltwater site compared to their freshwater counterparts. Catalase activity significantly increased in amphipods from the freshwater habitat after 1 and 3 h of heat exposure but decreased in their counterparts from the saltwater site after 3 h in response to the acute heat exposure (Fig. 4A).

##### Exposure to a gradual thermal challenge.

The baseline levels of catalase activity at 7 °C were significantly higher in amphipods from the saltwater site compared to their freshwater counterparts. Gradual temperature increase did not affect the activity of catalase in both of the studied populations of amphipods throughout the entire range of experimental temperatures (Fig. 4B).

#### Lactate content

##### Effect of acclimation at different temperatures.

The level of lactate was significantly lower in amphipods from the freshwater population, acclimated at 7 °C, compared to 15 °C. Acclimation at different temperatures had no significant effect on the lactate level in amphipods from the saltwater population (Fig. 2F).

##### Exposure to heat shock challenge.

The basal levels of lactate at 15 °C were significantly higher in amphipods from the freshwater population (Irkutsk) compared to saltwater Shira population. Acute thermal stress resulted in a significant increase of lactate level after 0.5 h and was increased until the end of the exposure (6 h) in amphipods from the saline like, whereas content of lactate significantly increased only after 3 h of acute thermal stress in amphipods from freshwater site (Fig. 4C).

##### Exposure to a gradual thermal challenge.

The basal levels of lactate at 7 °C were similar in amphipods from both studied populations. In amphipods from the freshwater Irkutsk population, gradual warming resulted in a significant elevation of lactate content at 17 °C that continued increasing until the end of the thermal exposure. In amphipods from Shira population, the content of lactate was constant within the temperature range of 7–29 °C and increased at 31 °C and 33 °C (Fig. 4D).

#### Glucose content

##### Effect of acclimation at different temperatures.

Acclimation at different temperatures had no significant effect on the glucose level in amphipods from the freshwater population. The level of glucose was significantly lower in amphipods from the freshwater population, acclimated at 7 °C, compared to 15 °C. (Fig. 2D).

##### Exposure to acute thermal challenge.

**Figure 5:**
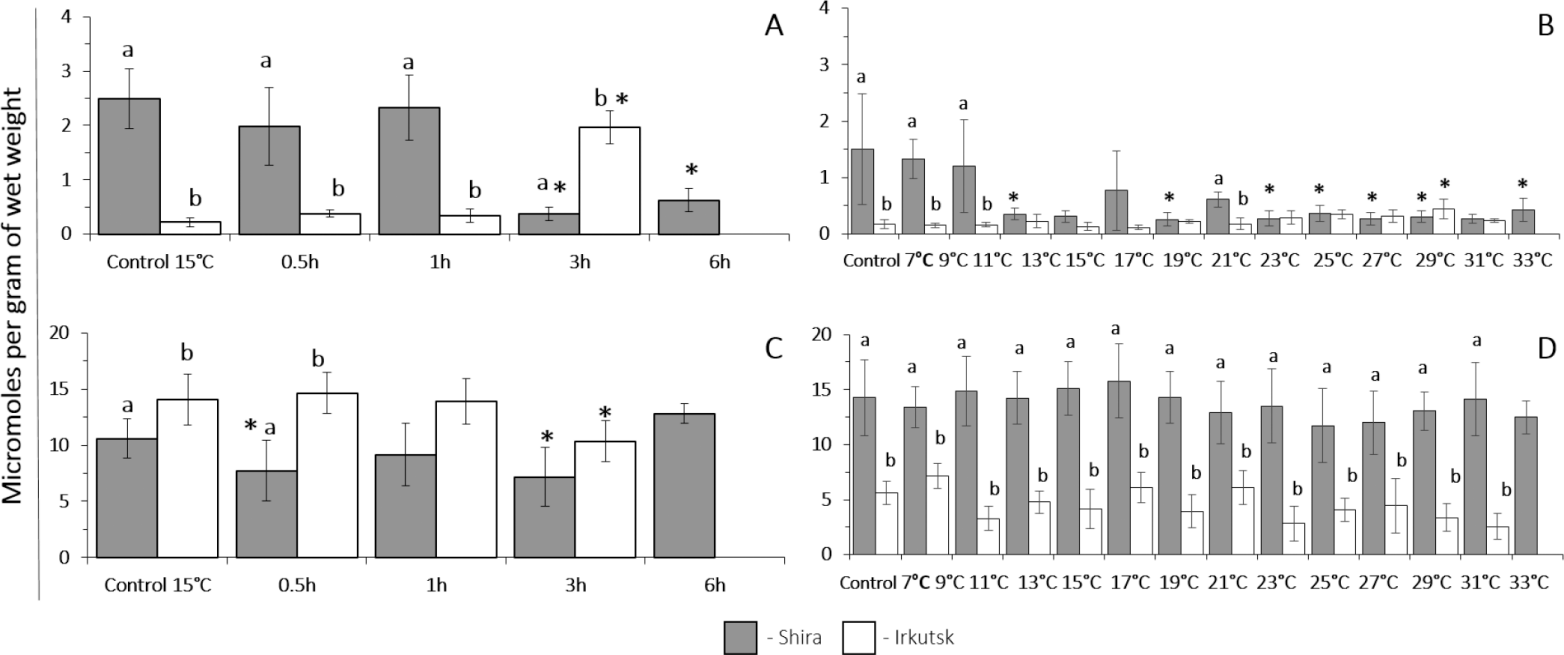
Glucose and glycogen content in amphipods *G. lacustris* from saltwater (Lake Shira) and freshwater (a lake in Irkutsk) populations. (A, B) glucose, (C, D) glycogen. (A, C) acute exposure to 30 °C (control 15 °C). (B, D) gradual warming (1 °C h^−1^) (control 7 °C). Different letters above the columns indicate differences between populations at a given time point. Asterisks (*) denote a significant change during the thermal exposure compared to the control animals from the same population (*P* < 0.05).

The basal level of glucose at 15 °C was 5-fold higher in amphipods from the saltwater site compared to their freshwater counterparts. In amphipods from the freshwater site, acute heat stress led to a significant increase in glucose content after 3 h. In contrast, acute thermal challenge resulted in a significant decrease of glucose content after 3 and 6 h in amphipods from a saline population (Fig. 5A).

##### Exposure to a gradual thermal challenge.

The basal level of glucose at 7 °C was higher in amphipods from the saltwater site compared to their freshwater counterparts (Fig. 5B). In amphipods from the freshwater population, glucose content was at the steady-state in the temperature range of 7°–27 °C and significantly increased at 29 °C. In amphipods from the saline lake, gradual warming resulted in a significant decline of glucose content at 13, 19, 23, 29 and 33 °C (Fig. 5B).

#### Glycogen content

##### Effect of acclimation at different temperatures.

The level of glycogen was significantly lower in amphipods from the freshwater population, acclimated at 7 °C, compared to 15 °C. Opposite to that, the level of glycogen was higher in amphipods from the saltwater population, acclimated at 7 °C compared to 15 °C (Fig. 2E).

##### Exposure to heat shock challenge.

The basal glycogen content at 15 °C was higher in amphipods from the freshwater population (Irkutsk) compared to the saltwater population (Lake Shira) (Fig. 5C). Acute heat stress resulted in a significant decrease of glycogen content in amphipods from the saltwater (after 0.5 and 3 h) and freshwater (after 3 h of exposure) populations.

##### Exposure to a gradual thermal challenge.

At 7 °C the basal levels of glycogen were significantly higher in amphipods from the saltwater site compared to their freshwater counterparts. Glycogen content did not change in freshwater or saltwater amphipods during the gradual temperature increase (Fig. 5D).

#### Adenylates

##### Effect of acclimation at different temperatures.

Acclimation at 7 and 15 °C had no significant effect on the levels of ATP, ADP and AMP in amphipods from the freshwater population; however, the level of ATP was significantly lower in amphipods from the saltwater population, acclimated at 7 °C, compared to 15 °C. (Figs. 2G, 2H and 2I).

##### Exposure to heat shock challenge.

**Figure 6:**
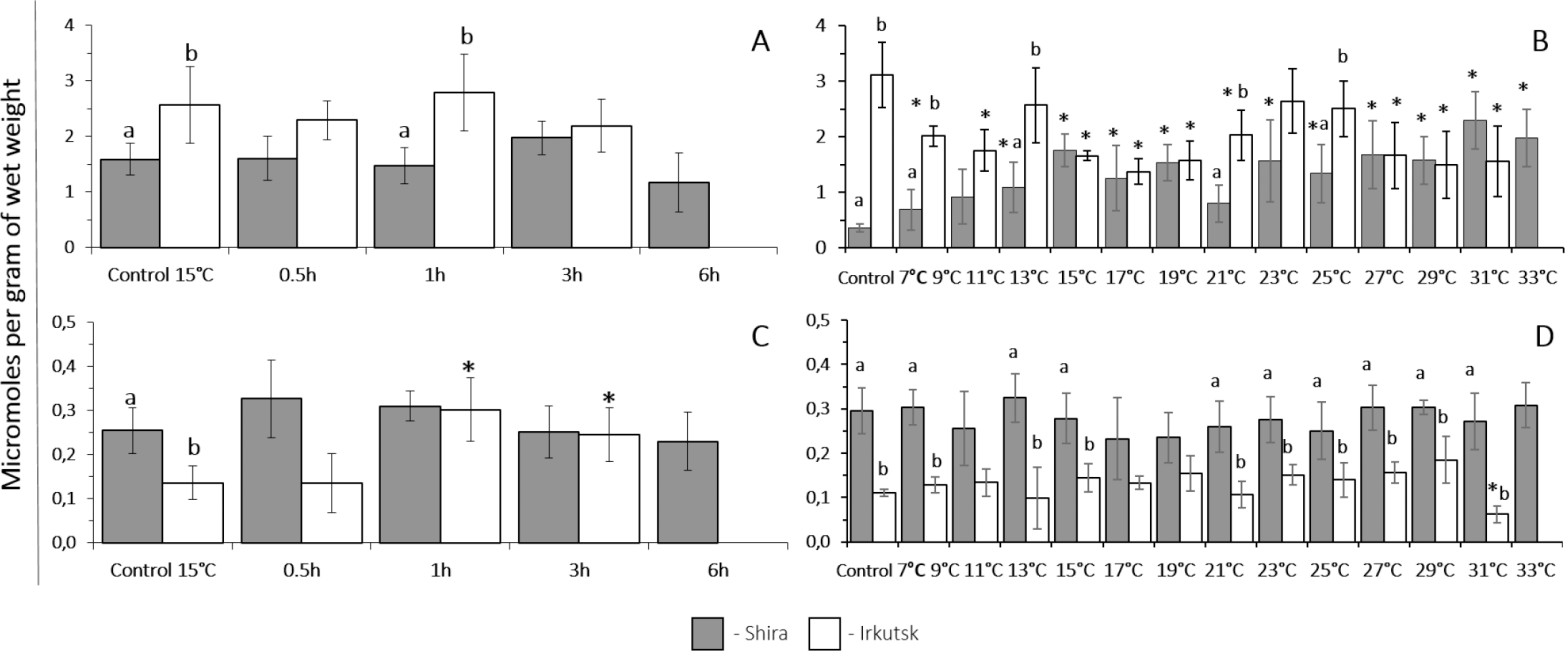
ATP and AMP levels in tissues of *G. lacustris* from saltwater (Lake Shira) and freshwater (a lake in Irkutsk) populations. (A, B) ATP, (C, D) AMP. (A, C) acute exposure to 30 °C (control 15 °C). (B, D) gradual warming (1 ° C h^−1^) (control 7 °C). Different letters above the columns indicate differences between populations at a given time point. Asterisks (*) denote a significant change during the thermal exposure compared to the control animals from the same population (*P* < 0.05).

**Figure 7:**
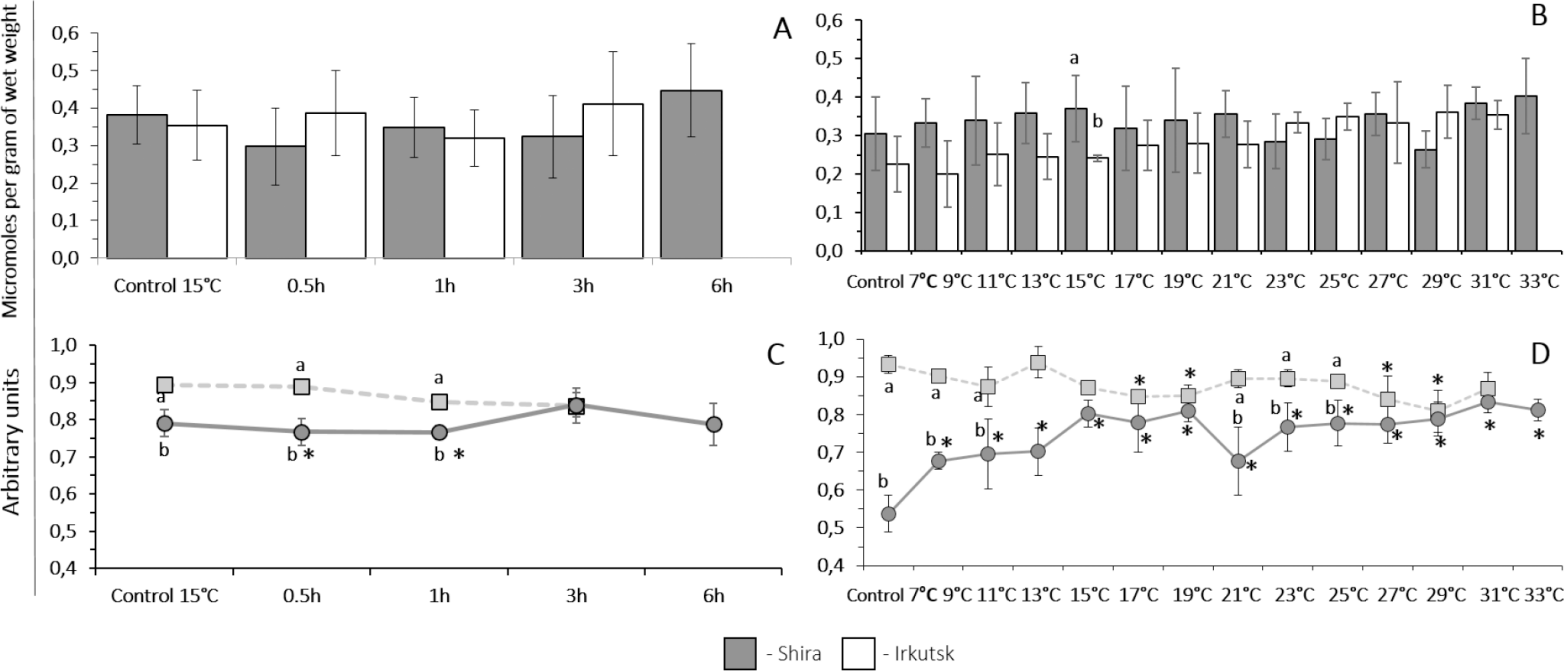
ADP and Adenylate energy charge (AEC) levels in tissues of *G. lacustris* from saltwater (Lake Shira) and freshwater (a lake in Irkutsk) populations. (A, B) ADP, (C, D) AEC. (A, C) acute exposure to 30 °C (control 15 °C). (B, D) gradual warming (1 °C h^−1^) (control 7 °C). Different letters above the columns indicate differences between populations at a given time point. Asterisks (*) denote a significant change during the thermal exposure compared to the control animals from the same population (*P* < 0.05).

The baseline of ATP content at 15 °C was significantly higher in amphipods from freshwater site compared to their saltwater counterparts (Fig. 6A). ATP level did not change in freshwater or saltwater amphipods throughout the gradual temperature increase. The basic level of AMP content at 15 °C was higher in amphipods from the saltwater Shira population compared to the freshwater Irkutsk population. AMP levels remained at the stead-state during acute heat stress in amphipods from the saline lake but increased after 1–3 h of exposure to 30 °C in amphipods from the freshwater site (Fig. 6C). Baseline ADP levels were similar in amphipods from both studied populations (Fig. 7A). There were no changes in the content of ADP in amphipods during acute heat stress.

##### Exposure to a gradual thermal challenge.

The baseline ATP content at 7 °C was significantly higher in amphipods from the freshwater population compared to the saltwater population. In amphipods from the freshwater site, gradual warming led to a decrease in tissue levels of ATP at the temperature of 9 °C or higher. In amphipods from the saltwater Shira site, gradual warming led to a steady increase in tissue ATP levels at the temperature of 13 °C or higher (Fig. 6B). In contrast to ATP, the baseline AMP content was higher in individuals from the saltwater population, compared to their freshwater counterparts. AMP level of freshwater population of amphipods was decreased at 31 °C in response to a gradual warming, but there were no changes in AMP content of Shira population (Fig. 6D). The baseline ADP content was similar in amphipods from the two studied sites. There were no changes in the ADP content in amphipods from both populations (Fig. 7B).

#### Adenylate energy charge

##### Effect of acclimation at different temperatures.

Acclimation at 7 °C and 15 °C had no significant effect on adenylate energy charge levels (AEC) in amphipods from the freshwater population; however, the value of AEC significantly decreased in amphipods from the saltwater population, acclimated at 7 °C, compared to 15 °C (Figs. 7C and 7D).

##### Exposure to heat shock challenge.

The baseline level of AEC at 15 °C was significantly higher in amphipods from the freshwater population compared to the saltwater population. Heat shock exposure led to a decrease of AEC in amphipods from the saltwater population already after 0.5 and 1 h, whereas no significant change in AEC of the freshwater population was observed (Fig. 7C).

##### Exposure to a gradual thermal challenge.

The baseline level of AEC after the acclimation at 7 °C was significantly higher in amphipods from the freshwater population. Gradual warming led to a significant decrease of AEC in amphipods of the freshwater population starting at 17 °C with a short leveling in the temperature range 21–25 °C and a following decrease at 27 and 29 °C. In contrast, the AEC in amphipods from saltwater population significantly increased during the gradual warming, starting from 9 °C, and the level of AEC stayed elevated during the whole exposure (Fig. 7D).

## Discussion

Our study shows that adaptation to different salinity regimes can influence thermotolerance and modulate key characteristics of cellular metabolism and stress responses in Holarctic amphipods *G. lacustris* Sars. Amphipods from a freshwater Irkutsk population were more sensitive to the thermal challenge experiencing higher mortality during acute and gradual warming compared to the amphipods from the saline Lake Shira. *G. lacustris* is well known for its broad salinity tolerance compared to its congeners such as *G. pulex* (Sutcliffe & Shaw, 1967). Earlier studies showed that freshwater *G. lacustris* can survive in 50 and 60% sea water for at least eight weeks, and regulate the hemolymph Na^+^ concentration until the media Na^+^ content reaches 250 mM L^−1^ (Sutcliffe & Shaw, 1967). Na^+^ concentration in the freshwater lake in Irkutsk (2.07 mM L^−1^) is significantly hypo-ionic with respect to *G. lacustris* hemolymph (that contains ∼150–200 mM Na^+^ under freshwater and brackish conditions, (Sutcliffe & Shaw, 1967), whereas the water in the saline Lake Shira is almost isoionic ([Na^+^] = 126 mM L^−1^) to *G. lacustris* hemolymph. This indicates that *G. lacustris* from the saline lake are less osmotically stressed than their freshwater counterparts and therefore spend less energy on ion- and osmoregulation which may explain their higher thermal tolerance compared to the freshwater population.

### Basal levels of cellular stress markers and energy metabolism

Bioenergetic indices (tissue levels of adenylates) measured under the temperature conditions similar to the mean habitat temperature and close to the physiological optimum for *G. lacustris* (15 °C), (Timofeyev, 2010), indicate more active aerobic metabolism in the amphipods from the freshwater Irkutsk population compared to their saltwater counterparts. Additionally, low levels of lactate in tissues of the saltwater amphipods suggest low rates of anaerobic metabolism (Pörtner, Heisler & Grieshaber, 1984; Livingstone, 1983). The freshwater amphipods appear to more strongly depend on anaerobic glycolysis compared to their saltwater counterparts as indicated by low glucose concentration and high levels of lactate, a major end product of anaerobic glycolysis in freshwater crustaceans (Pörtner, Heisler & Grieshaber, 1984; Livingstone, 1983).

Acclimation at a lower temperature (7 °C) resulted in a significant stimulation of activity of antioxidants in both saltwater and freshwater amphipod populations. This indicates that hypothermia induces oxidative stress in the amphipods, possibly reflecting higher oxygen solubility at low temperatures. Notably, the degree of stimulation of antioxidants was stronger in the saltwater Shira amphipods. Elevated activities of antioxidant enzymes and/or total antioxidant capacity, were also earlier reported from other euryhaline crustaceans acclimated or acclimatized to high salinities although profiles of antioxidant response to elevated salinity were tissue-specific (Henry et al., 2012; Wang et al., 2013; Romano & Zeng, 2012; Freire, Togni & Hermes-Lima, 2011; Li et al., 2015).

Energy metabolism of amphipods was also affected by the hypothermic conditions (7 °C). In saltwater Shira amphipods, the hypothermic acclimation (compared to the optimal temperature of 15 °C) led to a decrease in ATP, a slight decline in free glucose and accumulation of glycogen. This indicates stimulation of glycogen synthesis at low temperatures in the saltwater amphipods. In the freshwater population hypothermic exposure had opposite effects on energy metabolites leading to an increase in free glucose, decline in the glycogen reserves while the levels of adenylates remained unchanged. This suggests an increase in the glycolytic ATP production at 7 °C and may account for the maintenance of the steady-state ATP levels in hypothermia in the freshwater amphipods.

### Hyperthermic stress

Acute thermal challenge (30 °C) led to a significant increase in activities of all tested antioxidant enzymes (peroxidase, GST and catalase) in the freshwater population of amphipods. Elevated levels of antioxidant enzymes during the thermal challenge may reflect a temperature-induced increase in generation of reactive oxygen species (ROS) in the less tolerant freshwater population requiring upregulation of the cellular antioxidant capacity to protect the organism against oxidative stress. No such increase in antioxidant levels was found in the more thermotolerant saltwater population of amphipods. This may suggest that the existing capacity of antioxidant enzymes is sufficient to protect against the temperature-induced generation of ROS in these organisms and/or that their mitochondria do not increase the rate of ROS release during the heat exposure. Moreover, activity of antioxidant enzymes showed a transient but significant decline in response to the thermal challenge in *G. lacustris* from the saltwater site. This is unlikely to indicate a direct thermal damage to the antioxidant enzymes given high tolerance to heating in this population but may rather reflect a decrease in the ROS production at intermediate temperatures.

Gradual temperature increase starting from 7 °C resulted in a decrease of peroxidase activity in Shira population at 11–21 °C followed by a slight increase at 27–33 °C, which, however, did not reach the control levels. In the freshwater Irkutsk population, peroxidase activity declined at 19–21 °C then returned to the control levels. It is worth noting that the total peroxidase activity measured in this study encompasses activities of diverse cellular peroxidases such as peroxiredoxins, glutathione peroxidases, peroxisomal peroxidases and others. These enzymes are involved in a broad variety of cellular functions but they all play a role in the cellular redox balance and control of the oxidative stress (Mates, 2000; Espinosa-Diez et al., 2015; García-Triana & Yepiz-Plascencia, 2012). A decrease in peroxidase activity at intermediate temperatures (±6 °C around the optimum) may reflect either a decrease in ROS production, or serve as an energy-saving mechanism that redirects resources to other protective mechanisms (e.g., chaperones) to ensure optimal cellular protection at high temperatures. A similar decrease in activities of antioxidant enzymes has been previously shown in response to a variety of environmental stressors in amphipods (Timofeyev et al., 2008; Timofeyev et al., 2009). No changes in activity of catalase and glutathione S-transferase were found in the freshwater or saltwater populations of amphipods. Taken together, these findings indicate that baseline levels of antioxidant enzymes are sufficient to cope with the temperature-induced oxidative stress during gradual warming, and/or that unlike the acute thermal stress, gradual warming does not induce elevated ROS production in *G. lacustris*.

It is worth noting that our present study, while focusing on three key antioxidant enzymes, did not exhaustively test all cellular antioxidants that can be involved in protective responses against temperature and salinity challenge in aquatic organisms (Mates, 2000; Espinosa-Diez et al., 2015; García-Triana & Yepiz-Plascencia, 2012). Lower baseline levels of some antioxidants (such as catalase) and a lack of increase in antioxidant levels during heat stress in the saltwater amphipods may reflect the reliance of these organisms on other antioxidants, such as low molecular weight antioxidants obtained from the diet (Lesser, 2006). An earlier study showed that dietary antioxidants can strongly affect the degree and direction of the antioxidant response to environmental stressors in amphipods (Timofeyev et al., 2009). The differences in the predominant diet in amphipods from the saltwater and freshwater habitats could affect the tissue levels of non-enzymatic antioxidants. Thus, the main food source for the Lake Shira bentho-planktonic population of *G. lacustris* are freshly sedimented seston (Gladyshev et al., 2000; Degermendzhy et al., 2010) and planktonic copepods (Yemelyanova, Temerova & Degermendzhy, 2002) which are rich in low molecular weight antioxidants. In contrast, amphipods from the freshwater Irkutsk lake are benthic and primarily feed on detritus which is low in antioxidant content. This difference in diets may influence the oxidative stress response to heating in the two studied populations and requires further investigation. Regardless of the contribution of the low molecular weight antioxidants to the total antioxidant capacity and stress tolerance of *G. lacustris*, our data demonstrate that activation of enzymatic antioxidants is not involved in elevated heat tolerance of the saltwater amphipod population.

Metabolic responses to thermal challenge notably differed in the freshwater and saltwater populations of the studied amphipods. Significantly higher baseline levels of glucose in the saltwater population serves to provide energy resources during the acute thermal challenge (30 °C) until the 3 h of exposure when the level of glucose dropped about five-fold. Rapid accumulation of lactate indicates activation of anaerobic glycolysis as early as after 0.5 h of heat exposure in the saltwater population; this would provide additional ATP as the temperature-induced ATP demand of the tissues outstrips the aerobic ATP supply (Mathews, Van Holde & Ahern, 2000; Larade & Storey, 2002; Philp, MacDonald & Watt, 2005). The stable levels of ATP during the acute temperature stress in the saltwater amphipods indicates that the compensatory onset of anaerobic ATP production in combination with the aerobically produced ATP is sufficient to prevent ATP depletion. Significantly lower baseline levels of glucose in a freshwater population of amphipods indicates that this population may be energy-limited. Activation of glycogenolysis (as indicated by glycogen depletion and up to 5-fold glucose accumulation), and the onset of anaerobiosis (indicated by lactate accumulation) occurs much later in the freshwater amphipods (after 3 h of exposure), which is close to the LT100 for this population. Such a late transition to anaerobic metabolism and glycogenolysis indicate limited metabolic plasticity of the freshwater population during heat stress and may contribute to their higher sensitivity to temperature (and potentially other stressors).

Induction of anaerobiosis (as indicated by lactate accumulation) during the gradual warming occurred at considerably lower temperatures in the freshwater amphipods (17 °C) compared to their saltwater counterparts (31 °C). This indicates that the temperature-induced mismatch between the ATP demand and aerobic ATP supply occurs at lower temperatures in the less thermotolerant freshwater amphipods. Given the ability of saltwater amphipods to rapidly engage anaerobic pathways during acute thermal stress (30 °C), the delayed onset of anaerobiosis during the gradual temperature rise indicates that aerobic energy supply is sufficient to support ATP turnover until very high temperatures (31 °C) are reached. Transition to partial anaerobiosis during gradual warming is considered an indication of the upper critical temperatures of aerobic metabolism (*T*_*crit*_II) as which the aerobic scope of the organism disappears and only time-limited survival is possible (Sokolova et al., 2012; Bagwe, Beniash & Sokolova, 2015). The upper critical temperatures of aerobic metabolism (17 °C and 31 °C) are lower than the upper thermal limits (31 °C and 33 °C in the freshwater and the saltwater populations, respectively) and are considered a better indicator of the ecologically relevant thermal limits that determine biogeographic distribution of a species (Pörtner & Knust, 2007; Deutsch et al., 2015).

Even though anaerobic glycolysis is not engaged until 31 °C in the saltwater population of amphipods, a strong decrease in glucose content in the absence of glycogen accumulation at the temperatures at or above 15 °C indicate elevated catabolism of glucose. The most likely pathway for this catabolism is aerobic oxidation of glucose, which may explain an increase in ATP concentrations in saltwater amphipods during gradual warming. This indicates that the saltwater population maintains high cellular energy status over the most environmentally relevant temperature range. In contrast, the freshwater population of amphipods experiences energy deficiency during gradual warming indicated by a decline in ATP content and (at the extreme temperature of 31 °C) a decrease in AMP levels suggesting transamination of AMP.

No significant change in glycogen levels and only a slight increase of glucose level at 29 °C is observed during gradual warming in the freshwater population of amphipods. This finding indicates that glucose used for lactate production at elevated temperatures must be replenished by gluconeogenesis from other sources besides the glycogen (such as amino acids) in the freshwater amphipods. Generically, these findings indicate that a decrease in the aerobic scope during the gradual warming in the freshwater amphipods goes hand-in-hand with the impairment of the cellular energy status indicated by the decrease in ATP levels.

Overall, our data indicate that higher thermal sensitivity of the freshwater population of amphipods is associated with a lower baseline activity of antioxidant enzymes and a decreased ability to maintain energy balance and curb oxidative stress, compared to their saltwater counterparts, during exposure to acute and gradual temperature increase. High sensitivity of the freshwater population to warming was associated with energy limitations (indicated by low baseline glucose levels, downward shift of the critical temperature of aerobic metabolism and inability to maintain the steady-state ATP levels during warming), possibly reflecting a trade-off between the energy demands for osmoregulation and protection against the temperature stress. These findings suggest that freshwater populations of amphipods may be more vulnerable to the global climate change than those from saline habitats. On the other hand, brackish waters may serve as potential refuges during the climate change for eurysaline amphipod species such as *G. lacustris*.

##  Supplemental Information

10.7717/peerj.2657/supp-1 Data S1 Raw data on metabolites measurements

10.7717/peerj.2657/supp-2 Data S2 Raw data on enzymes measurements

10.7717/peerj.2657/supp-3 Data S3 Raw data on lactate measurements

10.7717/peerj.2657/supp-4 Data S4 Raw data on basic levels measurements

## Additional Information and Declarations

### Data Availability

The following information was supplied regarding data availability: The raw data has been supplied as a Supplementary File.
